# Diphtheria

**Published:** 1899-10-14

**Authors:** P. Watson Williams

**Affiliations:** Physician to Clifton College; and Physician in Charge of the Department for Diseases of the Throat, Bristol Royal Infirmary.


### Hospital Clinics and Medical Progress.

### DIPHTHERIA.

By P. Watson Williams, M.D.Lond., Physician to Clifton College; and Physician in Charge of the Department for Diseases of the Throat, Bristol Royal Infirmary.

(*Continued from page* 10.)

**Value of the Culture Method.**—One bacteriological examination is not sufficient to negative the clinical diagnosis of diphtheria, however skilfully the inoculation, incubation, and microscopical examination is conducted, and several negative separate examinations must be obtained before a case presenting clinical evidence in favour of diphtheria can be safely excluded from that category. Nevertheless, apart from inoculation of guinea pigs, the culture constitutes the most reliable diagnostic criterion we have, and the general reliability of cultures is well demonstrated by Sims Woodhead's[18] report on the bacteriological examination of 2,936 cases admitted on clinical grounds as diphtheria at the Metropolitan Asylums Board's hospitals. There were 6,408 examinations at the College of Physicians and Surgeons' laboratories, so that a considerable number of re-examinations were made, but in only 85 out of 2,936 cases did a re-examination reveal the presence of diphtheria bacilli where a negative diagnosis had been previously recorded. Again, out of 451 cases which were regarded as diphtheria in hospital, in 51 the presence of diphtheria bacilli could not be demonstrated; while out of 1,763 cases in which the diagnosis was doubtful, or was not stated, 1,112 were found to be true cases of diphtheria, 651 were apparently not diphtheria. Martin and Hunt,[19] at the University College Hospital, found the bacilli in 149 cases out of 167 admitted on clinical grounds as diphtheria; and in 18 cases no bacillus was found.

As the cases examined by Woodhead and Martin and Hunt were diagnosed clinically as diphtheria by experts, the value of the culture test is all the more obvious.

But negative results should not be considered to outweigh decided clinical evidence in favour of a diagnosis of diphtheria, at any rate until several different cultures have been made yielding uniformly negative evidence of a case being one of diphtheria. That this is the only safe course to adopt is well illustrated by Sims Woodhead's[20] statement that during the year 1896 there were examined at the laboratories of the Royal Colleges of Physicians and Surgeons of London 7,832 cases that had been certified as diphtheria, and of these 5,068 had diphtheria bacilli, but in the 1,764 in which no diphtheria bacilli were found there were 177 cases of paralysis, with 59 deaths—far too large a percentage to be caused by diseases other than diphtheria.

**Inoculation of Guinea Pigs** forms a reliable test, but is seldom resorted to except for the purpose of determining the virulence of a culture. Thus two or three minims of a blood-serum culture may be emulsified with sterile broth and inoculated under the skin of the abdomen. With virulent cultures the animal dies in from 30 hours to seven days; spreading gelatinous exudation at the seat of inoculation, serous effusions in the peritoneum, pleural cavity, and pericardium, and hæmorrhage into the adrenals are usual and characteristic features.

The guinea-pig is very susceptible to diphtheria, and therefore it is probably practically safe to regard a culture which is non-virulent to guinea-pigs as harmless in man, the more so as there is good reason to doubt that virulence to guinea-pigs is an absolute criterion of virulence to human beings.

**Pathological Anatomy.**—At the outset diphtheria is undoubtedly a local lesion, due to the growth of the Klebs-Loeffler bacillus in or on the epithelium of the mucous membrane, but at first producing only signs of simple inflammation. The earliest change is hyperæmia of the mucosa, with cloudy swelling of the epithelial cells. The diphtheria bacilli are present in greatest abundance in the more superficial granular parts of the exudation, but they are found in the epithelium and epithelial layers. The superficial layers of epithelium soon become necrosed, the dead epithelial cells being enclosed, together with the bacilli and leucocytes, in the meshes of fibrinous exudation. The resulting false membrane is usually firmly adherent, and can only be removed by detaching with it the inflamed and infiltrated epithelial layers, and inasmuch as the bacilli have penetrated deeper in the mucosa than the superficial portion removed with the false membrane, the process is renewed, and another false membrane soon forms on the denuded surface. On the other hand, only a thin superficial membrane, or no membrane whatever, may form, the tissues simply appearing red, inflamed, and swollen. False membranes were produced by Roger and Bayeux[21] in rabbits by the intratracheal injection of a solution of pure toxin. As those in which false membranes formed showed no signs of general toxæmia, while those in which no membrane formed died, they concluded that the formation of a false membrane showed a certain power of resistance to the poison.

The view till recently held that the bacilli are found only in the neighbourhood of the affected area is not absolutely correct, for they have been found in fatal cases in the lungs, spleen, and kidneys, &c., yet they are probably generally limited to the local lesion, and are always most numerous there. It is no longer doubtful that the bacilli there elaborate a toxin which, being absorbed in the circulation, produce the characteristic symptoms of the disease. Cultures from the blood of those dying early in diphtheria are usually sterile, but when the lung shows patches of broncho-pneumonia, diphtheria bacilli are always present in the blood (Park[22]). The toxin is a powerful poison, affecting especially the nerves; the heart, lungs, kidneys, and lymphatic glands also being affected.

As regards the nervous lesions, it would appear that certain slight and temporary changes occur in the nerve cells during the first 24 hours. The affected cells may either recover, or they may atrophy, with consequent degenerative changes of a Wallerian type and paralysis. Some of the cells recovering may account for the paralysis being usually incomplete. The paralysis is associated with parenchymatous degeneration of the myelin sheath affecting both sensory and motor elements. The work of Mouravjeff and of Crocq tends to show (by Nissle's method) that the primary alteration occurs in the cells of the anterior horn. But Martin showed that the parenchymatous degeneration at first affects the finer nerve branches of the peripheral nerves; and Batten[23] has reported that, although in five cases he observed the parenchymatous degeneration in various cranial nerves in the anterior and posterior roots and in the nerve fibres as they pass through the white matter to the grey of the spinal cord, yet he found the cells in the spinal cord and in the posterior root ganglia perfectly normal (Nissle's method).

The heart is very easily affected, and paralysis of the heart may occur early or late. It is possible that the early failure of the heart is due to a direct action of the toxin on the muscle; and Mott[24] has observed extreme early fatty degeneration of the heart, and yet no degeneration of the vagus nerve. But considering, as he does, that the toxin acts on the whole neuron, especially upon the terminal arborisation of the dendron and end flates, he explains the early cardiac failure by the effect of stress. The heart muscle and the respiratory muscles cannot rest, but even have to work harder, and therefore they succumb more readily to the action of the poison than the other muscles which are at rest, the cardiac muscle undergoing acute degenerative myocarditis.

Schemm[25] examined the heart muscle in 13 cases dying of diphtheria, eight having succumbed to cardiac paralysis. In all the cases he found fatty and granular degeneration, swelling and increase in the muscle nuclei, with hyaline degeneration and atrophy of the muscle fibres, but these changes were most pronounced in the cases dying from cardiac paralysis.

The blood undergoes certain changes, the most marked being a progressive leucocytosis, which subsides during convalescence. This increase in the number of the white corpuscles, which affects chiefly the finely granular oxyphile cells, has been differently interpreted in its clinical bearing. Kanthack and Lloydd,[26] from personal observations on this question, conclude that: (1) broadly speaking, a high leucocytosis signifies a good reaction, and was present in those that recovered; (2) a low leucocytosis at the height of the disease, before antitoxin has been injected, accompanies most, if not all fatal cases; (3) the high leucocytosis of well-reacting cases, after and during antitoxin treatment, steadily diminishes, the number of cells decreasing by 50 per cent. in three or four days.

J. S. Billings, jun.,[27] considers that his examination of the blood in 29 cases renders it improbable that any information of prognostic importance is to be gained by examination of the blood in diphtheria, though as a rule the amount of leucocytosis was directly proportionate to the severity of the case. The lungs may be affected with patches of broncho-pneumonia, and the bacilli of diphtheria have frequently been found in these patches, but in almost all cases other organisms, such as streptococci and staphylococci, are found in the patches, and the broncho-pneumonia is probably due to these cocci. The kidneys are usually pale and swollen, and epithelial degeneration, and necrosis and nephritis may be produced, the inflammatory changes being most pronounced in the glomeruli and tubercles. The urine sometimes contains Klebs-Loeffler bacilli. F. Smith[28] has found bacilli virulent in the urine of guinea pigs, and H. L. Barlow[29] found diphtheria bacilli in the urine of a patient, even after the urine had stood eight days at the room temperature. Others have found the bacillus post-mortem in the kidney, generally but not always associated with nephritis.

Albuminuria occurs in from 24 to 60 per cent. of the cases, but actual nephritis is probably rare and occurs in about 2 per cent. only.

The spleen is frequently enlarged, partly from vascular engorgement and partly from proliferative changes in the parenchyma.

[18] Lancet, June 11, 1896. [19] Brit. Med. Jour., Sept. 3, 1898. [20] Brit. Med. Jour., Sept. 3, 1898. [21] Exper. Diphth. Soc. de Biol., Press. Med., Mar. 17, 1897. [22] New York Med. Jour., Oct. 31, 1897. [23] Brit. Med. Jour., Nov. 19, 1898. [24] Brit. Med. Jour., Sept. 3, 1898. [25] Virch. Arch., Aug., 1890; Lancet, Jan. 25, 1896. [26] Allbutt's System, Vol. I. [27] New York Med. Rec., April 25, 1898. [28] Lancet, Nov. 19, 1898. [29] Lancet, Dec. 3, 1898.

